# Association of Gabapentinoids With the Risk of Opioid-Related Adverse Events in Surgical Patients in the United States

**DOI:** 10.1001/jamanetworkopen.2020.31647

**Published:** 2020-12-29

**Authors:** Katsiaryna Bykov, Brian T. Bateman, Jessica M. Franklin, Seanna M. Vine, Elisabetta Patorno

**Affiliations:** 1 Division of Pharmacoepidemiology and Pharmacoeconomics, Department of Medicine, Brigham and Women’s Hospital, Harvard Medical School, Boston, Massachusetts

## Abstract

**Importance:**

The use of gabapentinoids in multimodal postoperative analgesia is increasing; however, when coadministered with opioids, these drugs may potentiate central nervous system and respiratory depression.

**Objective:**

To evaluate the association between perioperative coadministration of gabapentinoids and opioids with inpatient opioid-related adverse events in surgical patients.

**Design, Setting, and Participants:**

This cohort study used propensity score trimming, stratification, and weighting of adults admitted for a major surgery between October 2007 and December 2017 who were treated with opioids on the day of surgery and included in the Premier Research database. Data analysis was conducted from February to April 2020.

**Exposure:**

Gabapentinoids (gabapentin or pregabalin) coadministered with opioids starting the day of surgery vs opioid therapy without gabapentinoids.

**Main Outcomes and Measures:**

Primary outcome was opioid overdose. Secondary outcomes included respiratory complications, unspecified adverse effects of opioid use, and a composite of these 3 outcomes. Patients were followed up for as long as 30 days from the day of surgery until deviation from the initial treatment regimen or discharge.

**Results:**

Gabapentinoids with opioids were administered to 892 484 of 5 547 667 eligible admissions (16.1%; mean [SD] age, 63.5 [11.8] years; 353 315 [39.6%] men). Among the 4 655 183 patients who received opioids only, the mean (SD) age was 63.7 (14.7) years, and 1 913 284 (41.1%) were men. Overall, 441 overdose events were identified, with absolute risks of 1.4 per 10 000 patients with gabapentinoid exposure and 0.7 per 10 000 patients receiving opioids only. Following propensity score trimming, the cohort included 737 383 patients exposed to gabapentinoids and 3 002 480 patients receiving opioids only. The primary analysis yielded the adjusted hazard ratio of 1.95 (95% CI, 1.49-2.55), and the number needed to treat for an additional overdose to occur was 16 914 patients (95% CI, 11 556-31 537 patients). Adjusted hazard ratios for secondary outcomes were 1.68 (95% CI, 1.59-1.78) for respiratory complications, 1.77 (95% CI, 1.61-1.93) for unspecified adverse effects of opioids, and 1.70 (95% CI, 1.62-1.79) for the composite outcome. The results were consistent across sensitivity analyses and subgroups identified by key clinical factors.

**Conclusions and Relevance:**

In this real-world cohort study of patients who underwent major surgery, concomitant use of gabapentinoids with opioids was associated with increased risk of opioid overdose and other opioid-related adverse events; however, the absolute risk of adverse events was low.

## Key Points

**Question:**

Is the adjuvant use of gabapentinoids with opioids associated with increased risk of opioid-related adverse events in patients undergoing surgery?

**Findings:**

In this cohort study of 5 547 667 US surgical admissions, the addition of gabapentinoids to opioids was associated with an increased risk of opioid overdose and other opioid-related adverse events; the absolute risk of adverse events was low.

**Meaning:**

These findings suggest that patients who receive gabapentinoids with opioids for postoperative analgesia should be closely monitored for possible respiratory depression.

## Teaser

This cohort study evaluates the association between perioperative coadministration of gabapentinoids and opioids with inpatient opioid-related adverse events in US surgical patients.

## Introduction

Amid the ongoing opioid epidemic in the United States, physicians are increasingly turning to multimodal pain management strategies. When effectively implemented, such strategies have been shown to improve the quality of care, decrease hospital stay, and reduce opioid use.^1,2,3^ In the postoperative setting, the 3 most common nonopioid pharmacologic analgesics used in multimodal pain management include acetaminophen, nonsteroidal anti-inflammatory drugs (NSAIDs), and gabapentinoids.^2^ While acetaminophen and NSAIDs have been coadministered with opioids for decades, the use of gabapentinoids is relatively recent.

Gabapentinoids (gabapentin and pregabalin) are anticonvulsant medications, commonly used for the treatment of chronic neuropathic pain. Their off-label use as adjuvant analgesics following surgery has been found to decrease postoperative pain and opioid consumption^4,5,6^ and is increasing. However, these drugs are associated with nontrivial adverse effects, including sedation and dizziness. Moreover, it has been reported that they can potentiate respiratory and central nervous system depression in an additive way when coadministered with opioids.^7^ In December 2019, the US Food and Drug Administration issued a warning on serious breathing difficulties associated with gabapentin and pregabalin in patients with other respiratory risk factors, including the use of opioids.^8^ Given the increasing use of gabapentinoids perioperatively and the lack of clinical data on their safety when coadministered with opioids for postoperative analgesia, we conducted a population-based cohort study in a large, nationwide database of US hospital admissions to assess whether coadministration of gabapentinoids with opioids is associated with increased risk of opioid-related adverse outcomes, including opioid overdose, in surgical patients.

## Methods

The research was approved by Partners Healthcare institutional review board. Given that data were deidentified, the need for informed consent was waived. A data use agreement was in place. This article complies with the Strengthening the Reporting of Observational Studies in Epidemiology (STROBE) reporting guideline and the Reporting of Studies Conducted using Observational Routinely-Collected Health Data (RECORD) guidelines for cohort studies as well as the guidelines specific to pharmacoepidemiological research (RECORD-PE).^9,10,11^

### Data Source and Study Cohort

The data for this investigation came from the Premier Research database, which contains data on approximately 20% of annual inpatient admissions in the United States. The database reflects all the information captured for billing purposes at the hospital level; data are routinely audited, verified, and validated. The hospitals included in the database are primarily nonprofit, nongovernmental, community, and teaching hospitals, and all patients treated at these hospitals are included in the database, independent of payer (Medicaid, Medicare, or commercial insurance).

The database includes deidentified patient demographic information; hospital characteristics; patient discharge diagnoses, captured via *International Classification of Diseases *(*ICD*) codes; and discharge status. It also includes date-stamped charges for all medications, procedures, and diagnostic tests done during inpatient admissions (ie, charge codes).

The study cohort included adults aged 18 years and older who underwent a major surgery between October 1, 2007, and December 31, 2017. The surgeries were defined based on *ICD-9* and *ICD-10* procedure codes and included hip or knee arthroplasty, coronary artery bypass grafting (CABG), cholecystectomy, colorectal resection, cystectomy, esophagectomy, gastrectomy, hysterectomy, laminectomy or spinal fusion, lobectomy, mastectomy, nephrectomy, pancreatectomy, or surgery for hip fracture or dislocation. We excluded patients with a discharge diagnosis of epilepsy or convulsions, given that these are nonpain-related indications for gabapentinoids, as well as patients with opioid use disorder or opioid dependence or with a code indicating pregnancy or labor and delivery. We further excluded patients who underwent multiple surgeries (ie, >1) during their hospital stay. Patients were required to have at least 1 charge code for an opioid medication on the day of surgery. To ensure the use of gabapentinoids for postoperative analgesia, we excluded patients who were exposed to gabapentinoids prior to the day of surgery. We further excluded patients who had a charge code for naloxone prior to the day of surgery to exclude patients who may have experienced opioid-related adverse events prior to surgery.

### Exposure Assessment

Exposure group assignment was based on medication charge codes on the day of surgery. All patients were required to have at least 1 charge code for an opioid (eTable 1 in the Supplement). Patients in the gabapentinoid group were required to have a charge code for either gabapentin or pregabalin. Patients in the reference group received opioids only. In the primary analysis, exposure was assessed on every day of follow-up, and patients were considered exposed through the day following a charge, ie, exposure was carried over through the next day, except when transdermal opioid patches were dispensed (following the day with a charge code, exposure lasted for 3 days for fentanyl and 7 days for buprenorphine patches). Patients in the gabapentinoid group could switch between gabapentin and pregabalin.

### Outcomes

The primary outcome was opioid overdose, defined based on a discharge diagnosis of overdose and a charge code for naloxone, an opioid antagonist used to reverse opioid-related respiratory depression. *ICD-9* codes used to define overdose, together with codes for poisoning by heroin, which were excluded in our definition, have been found to have a specificity of 99.9%.^12^ Secondary outcomes were respiratory complications (ie, acute respiratory failure, respiratory arrest, acute respiratory insufficiency or distress, dyspnea, asphyxia, hypoxemia, shortness of breath, and other pulmonary insufficiency or abnormalities); unspecified adverse effects of opioids (*ICD* diagnosis codes that included “adverse effect of opioids” or opioids “causing adverse effects in therapeutic use”) (eTable 2 in the Supplement); and a composite of opioid overdose, respiratory complications, or unspecified adverse effects of opioids. Outcome definitions are provided in eTable 2 in the Supplement.

For all outcomes, we required a charge code for naloxone in addition to a discharge diagnosis, and the timing of the outcome was set as the day of the naloxone charge. In our primary analysis, patients were followed up from the day of surgery for a maximum of 30 days until discharge or deviation from the initial treatment regimen (ie, gabapentinoids with opioids vs opioids alone) as defined on the day of surgery. Patients were censored on opioid discontinuation in either group, gabapentinoid discontinuation in the gabapentinoid group, or gabapentinoid initiation in the reference group.

### Covariates

A broad range of potential confounders and proxies for confounders were considered, including patient demographic characteristics (ie, age, sex, race [as reported on hospital billing form]), type of surgery and type of admission (ie, emergency, direct from clinic, nonemergency), hospital characteristics (ie, teaching status, urban vs rural, size, region), the year and quarter of hospitalization, patient chronic comorbid conditions (including pain-related conditions, cancer, depression, congestive heart failure, chronic obstructive pulmonary disease, and chronic kidney insufficiency), comorbidity score,^13^ inpatient use of other drugs on the day of surgery and prior to surgery (for patients who were admitted a day or more prior), and the type and the amount of opioids dispensed on the day of surgery. A complete list of covariates appears in eTable 3 in the Supplement. Total opioid dose received on the day of surgery was estimated in morphine milligram equivalents (MMEs) based on published conversion factors and equianalgesic opioid dosing tables.^14,15^ Patient chronic comorbid conditions were identified using discharge diagnoses and were assumed to be present, owing to their chronic nature, before admission. In addition, we assessed postoperative length of stay and estimated total MMEs dispensed on postoperative day 1.

### Statistical Analysis

Standardized differences were used to assess covariate balance between the group with gabapentinoid exposure and the reference group; meaningful imbalances were defined by an absolute standardized difference of more than 10%.^16^ Exposure propensity scores (PSs) were calculated for each patient as the estimated probability of receiving gabapentinoids on the day of surgery, conditional on all covariates (eTable 3 in the Supplement), and using a logistic regression model.^17^ To exclude noncomparable patients and reduce residual confounding, we conducted asymmetric trimming of the PS distributions by excluding patients with a PS value that corresponded to the 2.5th percentile or lower in the group with gabapentinoid exposure and to the 97.5th percentile or higher in the reference group.^18^ In the trimmed cohort, we created 50 strata based on the PS distribution in the group with gabapentinoid exposure and weighted patients who received opioids only proportional to the distribution of patients who received gabapentinoid with opioids in the stratum into which they fell.^19^ We assessed the balance of baseline characteristics in the weighted population and used weighted Cox proportional hazard regression models to estimate the adjusted hazard ratios (aHRs) between treatment with gabapentinoids and the risk of opioid-related adverse events.^19^ In addition, we estimated the number needed to treat (NNT) for 1 outcome to occur, which was derived from weighted risk difference, estimated using a generalized linear model with identity link function.

We conducted several sensitivity analyses to test the robustness of the primary results. First, to assess the potential for informative censoring, we observed patients until discharge (for a maximum of 30 days) according to their initial exposure group assignment on the day of surgery, irrespective of treatment changes, in an intention-to-treat approach. Second, to assess the potential effect of exposure misclassification, we conducted our primary analysis without exposure carryover, ie, patients were considered exposed only on days when they had treatment charges. Third, to exclude situations in which gabapentinoid initiation may have happened after an outcome on the day of surgery, we excluded patients with a naloxone charge on the day of surgery and started follow-up on postoperative day 1.

Finally, we conducted several subgroup analyses according to age (cut point, 65 years), sex, type of surgery (orthopedic procedures vs others), concomitant exposure to benzodiazepines on the day of surgery, and MMEs administered on the day of surgery. For MME subgroup analyses, we stratified our analyses based on median MME value in the overall cohort. In addition, we also evaluated gabapentin and pregabalin separately. In analyses specific to a gabapentinoid agent, patients were assigned to an exposure group based on their exposure on the day of surgery and were censored if they switched to a different gabapentinoid agent. For all sensitivity and subgroup analyses, PSs were reestimated, cohorts were retrimmed and reweighted, and postadjustment covariate balance was evaluated as described earlier.

All analyses were conducted using SAS version 9.4 (SAS Institute). No prespecified level of statistical significance was set.

## Results

### Study Population

**Figure 1.**
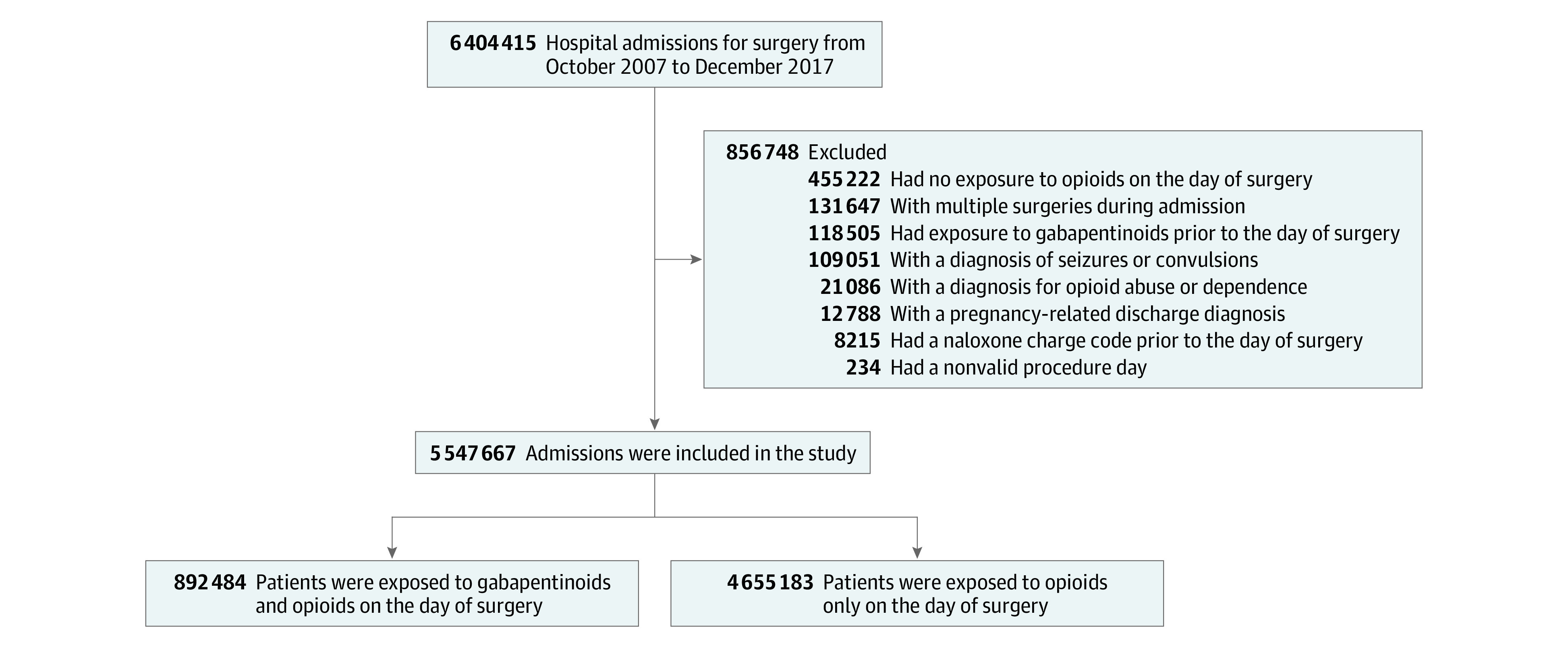
Study Cohort Exposure to medications was measured by medication charge codes.

Of 5 547 667 hospital admissions that met the inclusion criteria, 892 484 patients (16.1%; mean [SD] age, 63.5 [11.8] years; 353 315 [39.6%] men) were administered gabapentinoids in addition to opioids on the day of surgery (Figure 1). Among the 4 655 183 patients who received opioids only, the mean (SD) age was 63.7 (14.7) years, and 1 913 284 (41.1%) were men. Gabapentin was the more common agent, with 545 870 individuals (61.2%) in the gabapentinoid and opioid group receiving gabapentin; 313 243 (35.1%), pregabalin; and 33 371 (3.7%), both agents. Gabapentinoids were more likely to be administered following knee or hip arthroplasty and less likely to be administered for pain management of CABG, hysterectomy, or procedures for hips fractures (Table 1). The use of gabapentinoids increased substantially during the study period: 159 883 of 535 877 patients (29.8%) who underwent a surgery in 2017 received gabapentinoids compared with 23 414 of 379 677 (6.2%) during the first year of the study period (ie, October 2007 to September 2008). Mean (SD) opioid dose on the day of surgery was 277.1 (350.7) MMEs in the gabapentinoid-exposed group and 307.3 (366.3) MMEs in the reference group. The difference was smaller within subgroups of similar types of surgeries. Within orthopedic surgeries, the mean (SD) dose was 269.6 (388.0) MMEs in the gabapentinoid-exposed group and 272.5 (341.2) MMEs in the reference group. In patients who underwent nonorthopedic surgeries, mean (SD) dose was 369.6 (424.8) MMEs in the gabapentinoid-exposed group and 374.0 (406.5) MMEs in the reference group. 

**Table 1.** Selected Baseline Characteristics Before and After Propensity Score Adjustment

Characteristic	Patients, No. (%)			
	Unadjusted		Propensity score adjusted^a^	
	Opioids only (n = 4 655 183)	Gabapentinoids with opioids (n = 892 484)	Opioids only (n = 3 002 480)	Gabapentinoids with opioids (n = 737 383)
Demographic characteristics				
Age, mean (SD), y	63.7 (14.7)	63.5 (11.8)	63.6 (12.0)	63.6 (12.0)
Men	1 913 284 (41.1)	353 315 (39.6)	1 241 483 (41.3)	302 076 (41.0)
Race				
White	3 623 681 (77.8)	731 816 (82.0)	2 456 587 (81.8)	602 586 (81.7)
Black	403 615 (8.7)	73 439 (8.2)	241 575 (8.0)	60 131 (8.2)
Other^b^	627 887 (13.5)	87 229 (9.8)	304 318 (10.1)	74 666 (10.1)
Surgical characteristics				
Procedure				
Cholecystectomy	88 590 (1.9)	2096 (0.2)	3962 (0.1)	1326 (0.2)
Colorectal resection	365 300 (7.8)	17 616 (2.0)	52 120 (1.7)	15 768 (2.1)
Coronary artery bypass graft	430 558 (9.2)	7458 (0.8)	11 972 (0.4)	2461 (0.3)
Cystectomy	14 455 (0.3)	521 (0.1)	1156 (0.0)	388 (0.1)
Esophagectomy	7864 (0.2)	133 (0.0)	72 (0.0)	52 (0.0)
Gastrectomy	35 880 (0.8)	666 (0.1)	693 (0.0)	327 (0.0)
Hip arthroplasty	706 096 (15.2)	213 259 (23.9)	715 168 (23.8)	17 6347 (23.9)
Hysterectomy	391 276 (8.4)	13 616 (1.5)	27 691 (0.9)	9851 (1.3)
Knee arthroplasty	1 014 527 (21.8)	394 692 (44.2)	1 300 498 (43.3)	316 426 (42.9)
Laminectomy or spinal fusion	888 097 (19.1)	208 234 (23.3)	787 903 (26.2)	188 429 (25.6)
Lobectomy	58 581 (1.3)	3849 (0.4)	13 294 (0.4)	3345 (0.5)
Mastectomy	107 153 (2.3)	5840 (0.7)	17 633 (0.6)	4736 (0.6)
Nephrectomy	89 062 (1.9)	3870 (0.4)	10 017 (0.3)	2871 (0.4)
Pancreatectomy	14 888 (0.3)	612 (0.1)	1600 (0.1)	516 (0.1)
Surgery for hip fracture or dislocation	442 856 (9.5)	20 022 (2.2)	58 702 (2.0)	14 540 (2.0)
Total MMEs dispensed on the day of surgery, mean (SD)	307.3 (366.3)	277.1 (350.7)	283.8 (356.7)	283.2 (357.2)
Inpatient medications used on the day of surgery				
Anticonvulsant agents	84 534 (1.8)	27 705 (3.1)	69 761 (2.3)	17 739 (2.4)
Antidepressants	495 662 (10.6)	175 064 (19.6)	461 115 (15.4)	115 327 (15.6)
Antipsychotic agents	109 188 (2.3)	25 926 (2.9)	69 813 (2.3)	17 284 (2.3)
Benzodiazepines	3 601 887 (77.4)	742 998 (83.3)	2 499 824 (83.3)	611 754 (83.0)
Muscle relaxants	403 282 (8.7)	149 068 (16.7)	453 277 (15.1)	112 412 (15.2)
NSAIDs	1 349 137 (29.0)	365 260 (40.9)	1 178 390 (39.2)	290 888 (39.4)
Comorbidities				
Anxiety	440 787 (9.5)	119 444 (13.4)	344 814 (11.5)	85 398 (11.6)
Cancer	512 219 (11.0)	32 183 (3.6)	98 626 (3.3)	26 530 (3.6)
Congestive heart failure	316 395 (6.8)	30 430 (3.4)	89 835 (3.0)	22 156 (3.0)
COPD or asthma	774 092 (16.6)	159 483 (17.9)	490 891 (16.3)	120 767 (16.4)
Back and neck pain without neuropathic involvement	874 168 (18.8)	217 794 (24.4)	766 703 (25.5)	184 436 (25.0)
Diabetic neuropathy	61 807 (1.3)	26 202 (2.9)	41 781 (1.4)	11 253 (1.5)
Fibromyalgia	72 141 (1.5)	38 151 (4.3)	73 783 (2.5)	19 930 (2.7)
Other neuropathic pain	275 317 (5.9)	93 669 (10.5)	289 897 (9.7)	69 491 (9.4)
Comorbidity score, mean (SD)	1.0 (2.2)	0.4 (1.5)	0.3 (1.5)	0.3 (1.5)

Abbreviations: COPD, chronic obstructive pulmonary disease; MMEs, morphine milligram equivalents; NSAIDs, nonsteroidal anti-inflammatory drugs.

^a^ Propensity score adjustment included trimming patients from both tails of propensity score distribution, creating 50 strata based on the distribution among patients receiving gabapentinoids and opioids and weighting the patients in the reference group (opioids only). eTable 3 in the Supplement presents the full list of covariates and standardized differences.

^b^ Other included Hispanic, American Indian, Asian or Pacific Islander, and other, as reported on hospital billing forms.

After PS trimming, the cohort included 737 383 patients exposed to gabapentinoids and opioids and 3 002 480 patients exposed to opioids only (eFigure 1 in the Supplement). After PS weighting, all measured baseline characteristics, including MMEs, were well balanced between the 2 groups (Table 1). Postoperative length of stay was also well balanced (mean, 3.9 days; median, 4 days in each exposure group), ensuring equal opportunity to observe outcomes (eTable 4 in the Supplement).

### Opioid Overdose

In the overall population, we identified 441 overdose events, with absolute risks of 1.4 per 10 000 patients with gabapentinoid exposure and 0.7 per 10 000 patients receiving opioids only. Unadjusted results appear in eTable 5 in the Supplement. The adjusted HR was 1.95 (95% CI, 1.49-2.55), with an NNT for an additional overdose to occur of 16 914 patients (95% CI, 11 556-31 537 patients). Mean (SD) follow-up was 3.21 (1.61) days in the gabapentinoid-exposed group and 3.57 (1.77) days in the opioid-only group.

### Secondary Outcomes

The outcome of respiratory complications was identified in 15 177 patients (0.3%), while 4107 patients (<0.1%) had the outcome unspecified adverse effects of opioids, and 17 974 (0.3%) had the composite outcome of either overdose, respiratory complications, or unspecified adverse effects of opioids. Unadjusted results in the overall population appear in eTable 5 in the Supplement. The adjusted HRs for secondary outcomes were 1.68 (95% CI, 1.59-1.78) for respiratory complications, 1.77 (95% CI, 1.61-1.93) for unspecified adverse effects of opioids, and 1.70 (95% CI, 1.62-1.79) for the composite outcome (Table 2).

**Table 2.** Association Between Concomitant Exposure to Gabapentinoids and Opioids and Opioid-Related Adverse Events

Outcome	Events, No. (%)			
	Opioids only (n = 3 002 480)	Gabapentinoids with opioids (n = 737 383)	Adjusted hazard ratio (95% CI)	Adjusted NNT (95% CI)^a^
Primary outcome				
Overdose	205 (0.007)	94 (0.013)	1.95 (1.49-2.55)	16 914 (11 556-31 537)
Secondary outcomes				
Respiratory complications	5128 (0.17)	2001 (0.27)	1.68 (1.59-1.78)	994 (882-1139)
Unspecified adverse effects of opioids	1918 (0.06)	788 (0.11)	1.77 (1.61-1.93)	2327 (1962-2858)
Composite outcome^b^	6412 (0.21)	2531 (0.34)	1.70 (1.62-1.79)	771 (694-867)

Abbreviation: NNT, number needed to treat.

^a^ NNT for 1 adverse event to occur.

^b^ Composite outcome included overdose, respiratory complications, and unspecified adverse effects of opioids.

### Sensitivity and Subgroup Analyses

Intention-to-treat analyses yielded results that were consistent with the primary analysis findings, as did analyses in which exposure was not carried over to the next day following a medication charge (Table 3; eTable 6 in the Supplement). Starting follow-up on postoperative day 1 led to the exclusion of 83 610 patients who had a naloxone charge on the day of surgery and yielded similar findings, albeit with larger magnitude in the HR (Table 3).

**Table 3.** Sensitivity Analyses of the Association Between Concomitant Exposure to Gabapentinoids and Opioids and Opioid Overdose

Analysis	Events, No. (%)		Adjusted hazard ratio (95% CI)
	Opioids only (n = 3 002 480)	Gabapentinoids with opioids (n = 737 383)	
Primary analysis^a^	205 (0.007)	94 (0.013)	1.95 (1.49-2.55)
Intention-to-treat	228 (0.008)	96 (0.013)	1.72 (1.33-2.24)
No exposure carryover^b^	191 (0.006)	76 (0.010)	1.97 (1.47-2.65)
Follow-up from postoperative day 1, No./total No. (%)^c^	109/2 954 811 (0.004)	65/726 197 (0.009)	2.63 (1.86-3.72)

^a^ Follow-up started on the day of surgery; patients were considered exposed on the day of and through the day following a treatment charge and were censored on deviation from the initial treatment regimen.

^b^ Patients were considered exposed only on the day of a treatment charge.

^c^ Follow-up started on postoperative day 1. Patients with naloxone charge on the day of surgery were excluded; propensity scores were reestimated, and the population was reweighted.

**Figure 2.**
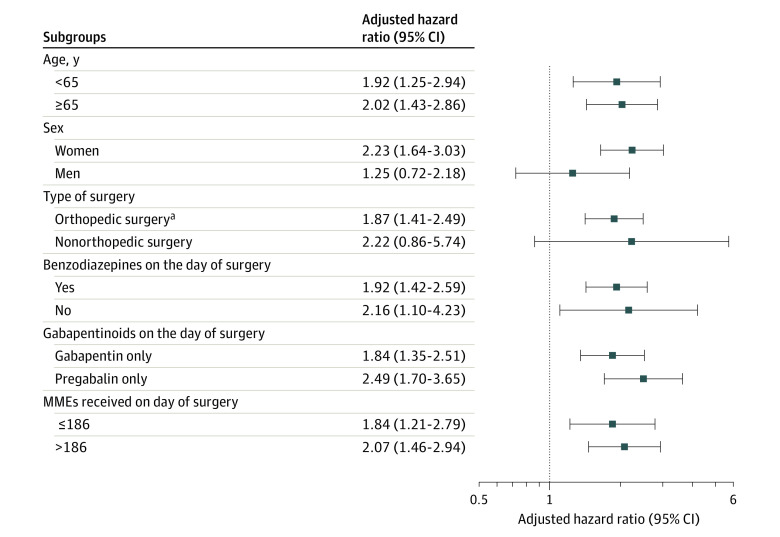
Association Between Concomitant Exposure to Gabapentinoids and Opioids and Opioid Overdose Among Subgroups MME indicates morphine milligram equivalent. ^a^Orthopedic surgeries included hip arthroplasty, knee arthroplasty, laminectomy or spinal fusion, and a surgery for hip fracture or dislocation.

Subgroup analyses had similar findings for individual gabapentinoid agents and found no evidence of effect modification by age, perioperative use of benzodiazepines, or opioid dose on the day of surgery (Figure 2; eFigure 2, eFigure 3, and eFigure 4 in the Supplement). In women, analysis of overdose yielded an adjusted HR of 2.23 (95% CI, 1.64-3.03), while in men, the association was lower, with an adjusted HR of 1.25 (95% CI, 0.72-2.18). Post hoc analyses showed no evidence of effect modification by sex (interaction between exposure and sex: adjusted HR, 1.66; 95% CI, 0.88-3.13). The results for the secondary outcomes were similar across the sex subgroups (eFigure 2, eFigure 3, and eFigure 4 in the Supplement).

## Discussion

In this large observational study of 5.5 million US hospital admissions for commonly performed surgeries, administration of gabapentinoids with opioids perioperatively was associated with increased risk of opioid-related adverse effects, including overdose, compared with pain management strategies that did not involve gabapentinoids. The overall occurrence of outcomes was very low, with overdoses occurring in less than 0.1% of patients.

Our findings add to the growing evidence that gabapentinoids can potentiate the respiratory depressant effects of opioids. Several observational studies have previously suggested that perioperative exposure to gabapentinoids is associated with increased risk of pulmonary complications.^20,21^ In a small randomized clinical trial, the combination of pregabalin and remifentanil behaved in an additive way to raise end-tidal carbon dioxide in healthy volunteers.^7^ Animal studies have shown that gabapentinoids can reverse tolerance to opioids and, subsequently, result in respiratory depression in mice.^22,23^ To our knowledge, our population-based study represents the largest evaluation of the association between the combination of gabapentinoids and opioids as part of multimodal postoperative pain management and opioid-related adverse events as they occur in real-world practice.

While we found that concomitant exposure to gabapentinoids and opioids was associated with increased risk of opioid-related adverse events during the postoperative period, the absolute risk of these events was extremely low. The low incidence may explain why previous meta-analyses of randomized clinical trials found sparse or no evidence of serious complications, such as respiratory depression, associated with the perioperative administration of gabapentinoids.^4,5,24^ It also highlights the importance of the overall benefit-risk assessment when considering adjuvant use of gabapentinoids, which have been shown to be associated with a decrease in postoperative pain and opioid consumption, although not consistently.^2,4,25^ A 2020 observational study^21^ found no association between gabapentinoids and reduced opioid consumption either on the day of or the day after total knee or hip arthroplasty. A randomized clinical trial that observed patients after discharge^25^ found that perioperative gabapentin had no effect on postoperative pain resolution, although it had some effect on promoting opioid cessation after surgery. In our cohort, the mean opioid dose on the day of surgery was slightly lower among patients receiving gabapentinoids than those in the reference group, although the difference was smaller once we stratified on the type of surgery (orthopedic vs other). Because of the nature of the Premier data, we could not evaluate the association of gabapentinoids with pain outcomes, either perioperatively or postoperatively, or the time to opioid cessation after discharge.

### Limitations

Our findings should be interpreted in the context of the limitations inherent in the nature of the health care data we used, given that the data were collected for billing purposes and not for research. First, while we rigorously attempted to identify and account for many potential confounders that could be differential between the exposure groups, the possibility of confounding due to unmeasured or incompletely captured factors cannot be completely ruled out. Second, in our outcome definitions, we had to rely on the coding of an event in the discharge abstract. Opioid-related adverse events may not have been coded as overdoses; we attempted to capture such cases via secondary outcomes. For the secondary outcome of respiratory complications, we did not have access to clinical parameters to confirm that events were related to opioids. However, we required a naloxone charge to reduce chances of outcome misclassification. Third, our exposure assessment was based on medication charge codes. We assumed a 2-day exposure risk window for charged medications. However, our sensitivity analyses varying exposure risk duration found very similar results. Limitations of this investigation also include the possibility of some imprecision in MME estimation because of the lack of standardized MME conversion for intravenous opioids or if not all the charged amount was used. However, the potential for opioid dose miscalculation is not expected to vary between exposure groups and, thus, is unlikely to substantially bias our findings. Furthermore, it is possible that patients who experienced opioid-related adverse events on the day of surgery were switched to gabapentinoids after the outcome. We could not establish temporality between outcomes and gabapentinoid exposure on the day of surgery. However, our sensitivity analysis, in which we excluded patients with outcomes on the day of surgery and started follow-up on postoperative day 1, found an even stronger association between gabapentinoids and opioid-related adverse events.

## Conclusions

The results of this study suggest that adjuvant use of gabapentinoids for pain management in postoperative setting is associated with increased risk of serious opioid-related adverse events, such as overdose and respiratory complications. The events were rare, corresponding to an NNT for an additional overdose to occur of more than 16 000 patients. Nevertheless, patients receiving multimodal pain management therapy that includes gabapentinoids should be closely monitored for possible respiratory depression.

## Notes

Supplement. **eTable 1.** Drug Exposure Definitions **eTable 2.** Outcome Definitions **eTable 3.** Baseline Patient Characteristics With Standardized Differences **eTable 4.** Variables Measured Following Exposure Group Assignment **eTable 5.** Unadjusted Associations Between Exposure to Gabapentinoids and Opioids and Opioid-Related Adverse Events in the Overall Population **eTable 6.** Primary and Sensitivity Analyses for Secondary Outcomes **eFigure 1.** Propensity Score Distribution Before and After Trimming and Weighting **eFigure 2.** Associations Between Exposure to Gabapentinoids and Opioids and the Outcome of Respiratory Complications Among Subgroups **eFigure 3.** Associations Between Exposure to Gabapentinoids and Opioids and the Outcome of Unspecified Adverse Effects of Opioids Among Subgroups **eFigure 4.** Associations Between Exposure to Gabapentinoids and Opioids and the Composite Outcome of Overdose, Respiratory Complications, and Unspecified Adverse Effects of Opioids Among Subgroups
