# Supplementary material for: Association of Gabapentinoids With the Risk of Opioid-Related Adverse Events in Surgical Patients in the United States
Source: JAMA Netw Open. 2020 Dec 29;3(12):e2031647. doi: 10.1001/jamanetworkopen.2020.31647 (PMC7772715; doi:10.1001/jamanetworkopen.2020.31647)
Supplement: Supplement. — eTable 1. Drug Exposure Definitions eTable 2. Outcome Definitions eTable 3. Baseline Patient Characteristics With Standardized Differences eTable 4. Variables Measured Following Exposure Group Assignment eTable 5. Unadjusted Associations Between Exposure to Gabapentinoids and Opioids and Opioid-Related Adverse Events in the Overall Population eTable 6. Primary and Sensitivity Analyses for Secondary Outcomes eFigure 1. Propensity Score Distribution Before and After Trimming and Weighting eFigure 2. Associations Between Exposure to Gabapentinoids and Opioids and the Outcome of Respiratory Complications Among Subgroups eFigure 3. Associations Between Exposure to Gabapentinoids and Opioids and the Outcome of Unspecified Adverse Effects of Opioids Among Subgroups eFigure 4. Associations Between Exposure to Gabapentinoids and Opioids and the Composite Outcome of Overdose, Respiratory Complications, and Unspecified Adverse Effects of Opioids Among Subgroups [file jamanetwopen-e2031647-s001.pdf]

Supplemental Online Content

Bykov K, Bateman BT, Franklin JM, Vine SM, Patorno E. Association of gabapentinoids with the risk of opioid-related adverse events in surgical patients in the United States. *JAMA Netw Open*. 2020;3(12):e2031647. doi:10.1001/jamanetworkopen.2020.31647

eTable 1. Drug Exposure Definitions

eTable 2. Outcome Definitions

eTable 3. Baseline Patient Characteristics With Standardized Differences

eTable 4. Variables Measured Following Exposure Group Assignment

eTable 5. Unadjusted Associations Between Exposure to Gabapentinoids and Opioids and Opioid-Related Adverse Events in the Overall Population

eTable 6. Primary and Sensitivity Analyses for Secondary Outcomes

eFigure 1. Propensity Score Distribution Before and After Trimming and Weighting

eFigure 2. Associations Between Exposure to Gabapentinoids and Opioids and the Outcome of Respiratory Complications Among Subgroups

eFigure 3. Associations Between Exposure to Gabapentinoids and Opioids and the Outcome of Unspecified Adverse Effects of Opioids Among Subgroups

eFigure 4. Associations Between Exposure to Gabapentinoids and Opioids and the Composite Outcome of Overdose, Respiratory Complications, and Unspecified Adverse Effects of Opioids Among Subgroups

This supplemental material has been provided by the authors to give readers additional information about their work.

© 2020 Bykov K et al. *JAMA Network Open.*

eTable 1. Drug Exposure Definitions

**Drug class**	**Generic names**
Opioid	alfentanil, buprenorphine, butorphanol, codeine, dihydrocodeine, fentanyl, hydrocodone, hydromorphone, levorphan, meperidine, methadone, morphine, oxycodone, oxymorphone, propoxyphene, pentazocine, remifentanil, sufentanil, tapentadol, tramadol
Gabapentinoid	gabapentin, pregabalin

© 2020 Bykov K et al. *JAMA Network Open.*

eTable 2. Outcome Definitions

Outcome	ICD-9 Definition	ICD-10 definition (last digit * stands for any, which will be A or D or S)
Opioid overdose	965.00 Poisoning by opium (alkaloids) unspecified<br>965.02 Poisoning by methadone<br>965.09 Poisoning by other opiates and related narcotics<br>E850.1 Accidental poisoning by methadone<br>E850.2Accidental poisoning by other opiates and related narcotics<br><br>**AND**<br>Charge code for naloxone	T400X1* Poisoning by opium, accidental<br>T400X2* intentional self-harm<br>T400X3* assault<br>T400X4* undetermined<br>T402X1* Poisoning by other opioids, accidental<br>T402X2* intentional self-harm<br>T402X3* assault<br>T402X4* undetermined<br>T403X1* Poisoning by methadone, accidental<br>T403X2* intentional self-harm<br>T403X3* assault<br>T403X4* undetermined<br>T404X1* Poisoning by other synthetic narcotics, accidental<br>T404X2* intentional self-harm<br>T404X3* assault<br>T404X4* undetermined<br>T40601* Poisoning by unspecified narcotics, accidental<br>T40602* intentional self-harm<br>T40603* assault<br>T40604* undetermined<br>T40691* Poisoning by other narcotics, accidental<br>T40692* intentional self-harm<br>T40693* assault<br>T40694* undetermined<br>**AND**<br>Charge code for naloxone
Respiratory complications	51881 Acute respiratory failure<br>51882 Other pulmonary insufficiency, not elsewhere classified<br>78603 Apnea<br>78605 Shortness of breath<br>78609 Other respiratory abnormalities<br>79901 Asphyxia<br>79902 Hypoxemia<br>7991 Respiratory arrest<br><br>**AND**<br>Charge code for naloxone	J9600 Acute respiratory failure, unspecified whether with hypoxia or hypercapnia<br>J9601 Acute respiratory failure with hypoxia<br>J9602 Acute respiratory failure with hypercapnia<br>J9690 Respiratory failure, unspecified, unspecified whether with hypoxia or hypercapnia<br>J9691 Respiratory failure, unspecified with hypoxia<br>J9692 Respiratory failure, unspecified with hypercapnia<br>J80 Acute respiratory distress syndrome<br>R0603 Acute respiratory distress<br>R0681 Apnea, not elsewhere classified<br>R0602 Shortness of breath<br>R0600 Dyspnea, unspecified<br>R0609 Other forms of dyspnea<br>R0689 Other abnormalities of breathing<br>R0901 Asphyxia<br>R0902 Hypoxemia<br>R092 Respiratory arrest<br>**AND**<br>Charge code for naloxone
Unspecified adverse	E9351 Methadone causing adverse effects in therapeutic use	T403X5* Adverse effect of methadone<br>T400X5* Adverse effect of opium<br>T402X5* Adverse effect of other opioids

© 2020 Bykov K et al. *JAMA Network Open.*

effects of opioids	E9352 Other opiates and related narcotics causing adverse effects in therapeutic use<br>**AND**<br>Charge code for naloxone	T404X5* Adverse effect of other synthetic narcotics<br>T40605* Adverse effect of unspecified narcotics<br>T40695* Adverse effect of other narcotics<br>**AND**<br>Charge code for naloxone

© 2020 Bykov K et al. *JAMA Network Open.*

eTable 3. Baseline Patient Characteristics With Standardized Differences

	Unadjusted			Propensity score adjusted		
	**Opioids only**	**Gabapentinoids + opioids**	**Std. diff. (%)**	**Opioids only**	**Gabapentinoids + opioids**	**Std. diff. (%)**
	N = 4,655,183	N = 892,484		N = 3,002,480	N = 737,383	
**Demographic characteristics**						
Age, mean (SD), y	63.7 (14.7)	63.5 (11.8)	-1.8	63.6 (12.0)	63.6 (12.0)	-0.2
Male sex - no. (%)	1913284 (41.1)	353315 (39.6)	-3.1	1241483 (41.3)	302076 (41.0)	-0.8
Race - no. (%)						
White	3623681 (77.8)	731816 (82.0)	10.4	2456587 (81.8)	602586 (81.7)	-0.3
Black	403615 (8.7)	73439 (8.2)	-1.6	241575 (8)	60131 (8.2)	0.4
Other	627887 (13.5)	87229 (9.8)	-11.6	304318 (10.1)	74666 (10.1)	0.0
**Surgical characteristics**						
Type of admission - no. (%)						
Non-emergency	3520865 (75.6)	672130 (75.3)	-0.8	2321564 (77.3)	567558 (77.0)	-0.8
Emergency	193241 (4.2)	3801 (0.4)	-25.1	6608 (0.2)	2113 (0.3)	1.3
Clinic	648664 (13.9)	198232 (22.2)	21.6	614556 (20.5)	153530 (20.8)	0.9
Other	292413 (6.3)	18321 (2.1)	-21.3	59753 (2.0)	14182 (1.9)	-0.5
Pre-procedure LOS, mean (SD), days	0.7 (2.2)	0.1 (0.7)	-37.2	0 (0.3)	0 (0.2)	-4.3
**Procedure - no. (%)**						
Cholecystectomy	88590 (1.9)	2096 (0.2)	-16.3	3962 (0.1)	1326 (0.2)	1.2
Colorectal resection	365300 (7.8)	17616 (2.0)	-27.4	52120 (1.7)	15768 (2.1)	2.9
Coronary artery bypass graft	430558 (9.2)	7458 (0.8)	-39.2	11972 (0.4)	2461 (0.3)	-1.1
Cystectomy	14455 (0.3)	521 (0.1)	-5.9	1156 (0)	388 (0.1)	0.7
Esophagectomy	7864 (0.2)	133 (0.0)	-5.1	72 (0.0)	52 (0.0)	0.7
Gastrectomy	35880 (0.8)	666 (0.1)	-10.7	693 (0.0)	327 (0.0)	1.2
Hip arthroplasty	706096 (15.2)	213259 (23.9)	22.1	715168 (23.8)	176347 (23.9)	0.2
Hysterectomy	391276 (8.4)	13616 (1.5)	-32.1	27691 (0.9)	9851 (1.3)	3.9
Knee arthroplasty	1014527 (21.8)	394692 (44.2)	49.1	1300498 (43.3)	316426 (42.9)	-0.8
Laminectomy or spinal fusion	888097 (19.1)	208234 (23.3)	10.4	787903 (26.2)	188429 (25.6)	-1.6
Lobectomy	58581 (1.3)	3849 (0.4)	-9.0	13294 (0.4)	3345 (0.5)	0.2
Mastectomy	107153 (2.3)	5840 (0.7)	-13.7	17633 (0.6)	4736 (0.6)	0.7
Nephrectomy	89062 (1.9)	3870 (0.4)	-13.8	10017 (0.3)	2871 (0.4)	0.9
Pancreatectomy	14888 (0.3)	612 (0.1)	-5.7	1600 (0.1)	516 (0.1)	0.7
Surgery for hip fracture or dislocation	442856 (9.5)	20022 (2.2)	-31.3	58702 (2.0)	14540 (2.0)	0.1
**Type of opioid on the day of surgery - no. (%)**						

© 2020 Bykov K et al. *JAMA Network Open.*

Oxycodone	1256948 (27.0)	491374 (55.1)	59.5	1520293 (50.6)	373251 (50.6)	0.0
Morphine	2546617 (54.7)	411980 (46.2)	-17.2	1465881 (48.8)	356482 (48.3)	-1.0
Meperidine	436666 (9.4)	58281 (6.5)	-10.5	213250 (7.1)	51655 (7.0)	-0.4
Hydromorphone	2554461 (54.9)	532919 (59.7)	9.8	1828710 (60.9)	446088 (60.5)	-0.8
Hydrocodone	660566 (14.2)	142143 (15.9)	4.9	505744 (16.8)	121919 (16.5)	-0.8
Fentanyl	3742110 (80.4)	717097 (80.3)	-0.1	2426260 (80.8)	594835 (80.7)	-0.4
Tramadol	168638 (3.6)	94116 (10.5)	27.2	201755 (6.7)	51528 (7.0)	1.1
Other	376316 (8.1)	66553 (7.5)	-2.3	230111 (7.7)	55303 (7.5)	-0.6
Total MMEs on the day of surgery, mean (SD)	307.3 (366.3)	277.1 (350.7)	-8.4	283.8 (356.7)	283.2 (357.2)	-0.2
Intravenous opioids	4520470 (97.1)	851604 (95.4)	-8.9	2886162 (96.1)	708116 (96.0)	-0.5
**Medication use on the day of surgery - no. (%)**						
Anticonvulsant agents	84534 (1.8)	27705 (3.1)	8.3	69761 (2.3)	17739 (2.4)	0.5
Antidepressants	495662 (10.6)	175064 (19.6)	25.2	461115 (15.4)	115327 (15.6)	0.8
Antipsychotic agents	109188 (2.3)	25926 (2.9)	3.5	69813 (2.3)	17284 (2.3)	0.1
Barbiturates	1674 (0.0)	187 (0.0)	-0.9	690 (0.0)	157 (0.0)	-0.1
Benzodiazepines	3601887 (77.4)	742998 (83.3)	14.8	2499824 (83.3)	611754 (83)	-0.8
Dementia medications	66635 (1.4)	7713 (0.9)	-5.3	23086 (0.8)	5602 (0.8)	-0.1
Hypnotic agents	311169 (6.7)	53838 (6.0)	-2.7	170011 (5.7)	41710 (5.7)	0.0
Lithium	6360 (0.1)	1994 (0.2)	2.0	5735 (0.2)	1393 (0.2)	0.0
Muscle relaxants	403282 (8.7)	149068 (16.7)	24.3	453277 (15.1)	112412 (15.2)	0.4
NSAIDs	1349137 (29)	365260 (40.9)	25.2	1178390 (39.2)	290888 (39.4)	0.4
**Comorbidities - no. (%)**						
Alcohol abuse or dependence	102626 (2.2)	12984 (1.5)	-5.6	43035 (1.4)	10522 (1.4)	-0.1
Anxiety	440787 (9.5)	119444 (13.4)	12.3	344814 (11.5)	85398 (11.6)	0.3
Bipolar disorder	51543 (1.1)	15033 (1.7)	4.9	39082 (1.3)	9867 (1.3)	0.3
Cancer	512219 (11.0)	32183 (3.6)	-28.7	98626 (3.3)	26530 (3.6)	1.7
Chronic renal insufficiency	364751 (7.8)	54252 (6.1)	-6.9	163872 (5.5)	40323 (5.5)	0.0
Congestive heart failure	316395 (6.8)	30430 (3.4)	-15.4	89835 (3.0)	22156 (3.0)	0.1
COPD or asthma	774092 (16.6)	159483 (17.9)	3.3	490891 (16.3)	120767 (16.4)	0.1
Dementia	220705 (4.7)	12440 (1.4)	-19.5	37997 (1.3)	9565 (1.3)	0.3
Depression	565720 (12.2)	152551 (17.1)	14.0	451486 (15)	111117 (15.1)	0.1
Diabetes	990112 (21.3)	206169 (23.1)	4.4	627837 (20.9)	154147 (20.9)	0.0
Liver disease	140867 (3.0)	19612 (2.2)	-5.2	58621 (2.0)	14681 (2.0)	0.3
Psychosis	40670 (0.9)	5007 (0.6)	-3.7	15232 (0.5)	3814 (0.5)	0.1
Back and neck pain without neuropathic involvement	874168 (18.8)	217794 (24.4)	13.7	766703 (25.5)	184436 (25)	-1.2
Diabetic neuropathy	61807 (1.3)	26202 (2.9)	11.2	41781 (1.4)	11253 (1.5)	1.1
Fibromyalgia	72141 (1.5)	38151 (4.3)	16.3	73783 (2.5)	19930 (2.7)	1.5

© 2020 Bykov K et al. *JAMA Network Open.*

Postherpetic neuralgia	1606 (0.0)	884 (0.1)	2.5	1312 (0.0)	375 (0.1)	0.3
Other neuropathic pain	275317 (5.9)	93669 (10.5)	16.8	289897 (9.7)	69491 (9.4)	-0.8
Osteoarthritis	1718672 (36.9)	597969 (67)	63.1	1976068 (65.8)	482807 (65.5)	-0.7
Rheumatoid arthritis	111347 (2.4)	32887 (3.7)	7.5	98064 (3.3)	24010 (3.3)	-0.1
Other arthritis, arthropathies and musculoskeletal pain	593161 (12.7)	131643 (14.8)	5.8	450616 (15.0)	109151 (14.8)	-0.6
Combined comorbidity score, mean (SD)	1.0 (2.2)	0.4 (1.5)	-30.9	0.3 (1.5)	0.3 (1.5)	1.2
**Inpatient use of medication before surgery - no. (%)**						
Anticonvulsant agents	24474 (0.5)	449 (0.1)	-8.9	942 (0.0)	180 (0.0)	-0.4
Antidepressants	159342 (3.4)	3055 (0.3)	-22.8	6916 (0.2)	1238 (0.2)	-1.4
Antipsychotic agents	47864 (1.0)	653 (0.1)	-12.9	1123 (0.0)	226 (0.0)	-0.4
Barbiturates	726 (0.0)	5 (0.0)	-1.7	4 (0.0)	1 (0.0)	0.0
Benzodiazepines	492798 (10.6)	9801 (1.1)	-41.3	24057 (0.8)	4710 (0.6)	-1.9
Dementia medications	39145 (0.8)	400 (0.0)	-12.0	383 (0.0)	65 (0.0)	-0.4
Hypnotic agents	84860 (1.8)	1499 (0.2)	-16.7	3540 (0.1)	598 (0.1)	-1.2
Lithium	1297 (0.0)	28 (0.0)	-2.0	70 (0.0)	16 (0.0)	0.0
Muscle relaxants	55763 (1.2)	2179 (0.2)	-11.3	6909 (0.2)	1309 (0.2)	-1.2
NSAIDs	102626 (2.2)	5615 (0.6)	-13.4	17280 (0.6)	4159 (0.6)	-0.2
Opioids	986666 (21.2)	31111 (3.5)	-55.9	85452 (2.8)	20052 (2.7)	-0.8
**Hospital characteristics - no. (%)**						
Region						
Northeast	702680 (15.1)	139481 (15.6)	1.5	483416 (16.1)	115323 (15.6)	-1.3
South	2165560 (46.5)	390788 (43.8)	-5.5	1325232 (44.1)	326867 (44.3)	0.4
Midwest	905863 (19.5)	181127 (20.3)	2.1	601615 (20.0)	148633 (20.2)	0.3
West	881080 (18.9)	181088 (20.3)	3.4	592217 (19.7)	146560 (19.9)	0.4
Number of beds						
000-099	168049 (3.6)	42231 (4.7)	5.6	135052 (4.5)	33490 (4.5)	0.2
100-199	548080 (11.8)	124547 (14)	6.5	435261 (14.5)	106699 (14.5)	-0.1
200-299	780745 (16.8)	153254 (17.2)	1.1	520496 (17.3)	128213 (17.4)	0.1
300-399	876128 (18.8)	151675 (17.0)	-4.8	529583 (17.6)	129732 (17.6)	-0.1
400-499	748654 (16.1)	135213 (15.2)	-2.6	449842 (15.0)	108639 (14.7)	-0.7
500+	1533527 (32.9)	285564 (32.0)	-2.0	932246 (31.0)	230610 (31.3)	0.5
Teaching Hospital	2004246 (43.1)	406377 (45.5)	5.0	1328736 (44.3)	325491 (44.1)	-0.2
Rural Hospital	462378 (9.9)	84877 (9.5)	-1.4	287198 (9.6)	69390 (9.4)	-0.5
**Admission Year_Quarter - no. (%)**						
2007_4	89688 (1.9)	5085 (0.6)	-12.2	18050 (0.6)	4522 (0.6)	0.2
2008_1	90192 (1.9)	5870 (0.7)	-11.3	21205 (0.7)	5364 (0.7)	0.3
2008_2	89910 (1.9)	6315 (0.7)	-10.7	23029 (0.8)	5744 (0.8)	0.1

© 2020 Bykov K et al. *JAMA Network Open.*

2008_3	86473 (1.9)	6144 (0.7)	-10.4	22361 (0.7)	5531 (0.8)	0.1
2008_4	87084 (1.9)	6544 (0.7)	-10.0	24204 (0.8)	5940 (0.8)	0.0
2009_1	95519 (2.1)	7796 (0.9)	-9.8	29130 (1.0)	7187 (1.0)	0.0
2009_2	94537 (2.0)	8004 (0.9)	-9.5	29934 (1.0)	7360 (1.0)	0.0
2009_3	94850 (2.0)	8223 (0.9)	-9.3	30789 (1.0)	7635 (1.0)	0.1
2009_4	96208 (2.1)	8485 (1.0)	-9.2	31923 (1.1)	7898 (1.1)	0.1
2010_1	110148 (2.4)	10062 (1.1)	-9.5	38170 (1.3)	9356 (1.3)	0.0
2010_2	109017 (2.3)	10314 (1.2)	-9.1	39110 (1.3)	9633 (1.3)	0.0
2010_3	108572 (2.3)	10551 (1.2)	-8.8	40100 (1.3)	9799 (1.3)	-0.1
2010_4	113821 (2.4)	11620 (1.3)	-8.4	44426 (1.5)	10891 (1.5)	0.0
2011_1	129155 (2.8)	13725 (1.5)	-8.5	52615 (1.8)	12850 (1.7)	-0.1
2011_2	125736 (2.7)	14025 (1.6)	-7.8	53719 (1.8)	13165 (1.8)	0.0
2011_3	123703 (2.7)	14205 (1.6)	-7.4	54494 (1.8)	13359 (1.8)	0.0
2011_4	124653 (2.7)	15333 (1.7)	-6.5	59000 (2.0)	14431 (2.0)	-0.1
2012_1	131380 (2.8)	17910 (2.0)	-5.3	69442 (2.3)	16849 (2.3)	-0.2
2012_2	126418 (2.7)	18198 (2.0)	-4.4	70033 (2.3)	17085 (2.3)	-0.1
2012_3	121260 (2.6)	18078 (2.0)	-3.9	69070 (2.3)	16918 (2.3)	0.0
2012_4	131044 (2.8)	20429 (2.3)	-3.3	78666 (2.6)	19184 (2.6)	-0.1
2013_1	130151 (2.8)	21373 (2.4)	-2.5	82260 (2.7)	19967 (2.7)	-0.2
2013_2	128533 (2.8)	21764 (2.4)	-2.0	83364 (2.8)	20133 (2.7)	-0.3
2013_3	124371 (2.7)	22281 (2.5)	-1.1	84688 (2.8)	20487 (2.8)	-0.3
2013_4	128761 (2.8)	24688 (2.8)	0.0	93722 (3.1)	22651 (3.1)	-0.3
2014_1	128139 (2.8)	26430 (3.0)	1.3	98102 (3.3)	23829 (3.2)	-0.2
2014_2	127854 (2.7)	27648 (3.1)	2.1	101096 (3.4)	24604 (3.3)	-0.2
2014_3	127506 (2.7)	28263 (3.2)	2.5	102538 (3.4)	24939 (3.4)	-0.2
2014_4	130646 (2.8)	30883 (3.5)	3.8	111401 (3.7)	27120 (3.7)	-0.2
2015_1	130571 (2.8)	31491 (3.5)	4.1	112469 (3.7)	27423 (3.7)	-0.1
2015_2	127994 (2.7)	31904 (3.6)	4.7	112165 (3.7)	27342 (3.7)	-0.1
2015_3	124661 (2.7)	33428 (3.7)	6.1	113455 (3.8)	27891 (3.8)	0.0
2015_4	121914 (2.6)	37600 (4.2)	8.8	122826 (4.1)	30306 (4.1)	0.1
2016_1	121293 (2.6)	38767 (4.3)	9.5	124620 (4.2)	30641 (4.2)	0.0
2016_2	117894 (2.5)	38579 (4.3)	9.9	121443 (4.0)	29917 (4.1)	0.1
2016_3	113004 (2.4)	38675 (4.3)	10.6	117449 (3.9)	29033 (3.9)	0.1
2016_4	116529 (2.5)	41911 (4.7)	11.8	125101 (4.2)	30947 (4.2)	0.2
2017_1	108139 (2.3)	42658 (4.8)	13.3	116424 (3.9)	29301 (4.0)	0.5
2017_2	100572 (2.2)	42523 (4.8)	14.3	105167 (3.5)	26606 (3.6)	0.6
2017_3	87684 (1.9)	38239 (4.3)	13.9	89045 (3.0)	22305 (3.0)	0.3
2017_4	79599 (1.7)	36463 (4.1)	14.2	85675 (2.9)	21240 (2.9)	0.2

© 2020 Bykov K et al. *JAMA Network Open.*

Admission Season						
December - January	800763 (17.2)	148980 (16.7)	-1.4	507743 (16.9)	124583 (16.9)	0.0
June - July	761248 (16.4)	146150 (16.4)	0.1	491069 (16.4)	120442 (16.3)	-0.1

Abbreviations: COPD, chronic obstructive pulmonary disease; LOS, length of stay; MMEs, morphine milligram equivalents; NSAIDs, nonsteroidal anti-inflammatory drugs; std diff, standardized difference

© 2020 Bykov K et al. *JAMA Network Open.*

eTable 4. Variables Measured Following Exposure Group Assignment

Characteristic	Opioids only	Gabapentinoids + opioids
	N = 3,002,480	N = 737,383
Post-procedure length of stay (including procedure day)		
mean (SD), days	3.9 (2.4)	3.9 (2.2)
median (IQR), days	4 (3 – 4)	4 (3 – 4)
Total MMEs dispensed on postoperative day 1		
mean (SD)	88.4 (113.3)	85.3 (113.7)
median (IQR)	58.5 (25.0 – 105.0)	52.5 (22.5 – 100.0)

IQR, interquartile range, 25th – 75th percentile; MMEs, morphine milligram equivalents; SD, standard deviation
Characteristics were assessed in the PS-weighted study population but were not included in the PS estimation.

© 2020 Bykov K et al. *JAMA Network Open.*

eTable 5. Unadjusted Associations Between Exposure to Gabapentinoids and Opioids and Opioid-Related Adverse Events in the Overall Population

	Opioids only	Gabapentinoids + with opioids	Hazard Ratios (95% CIs)
**Outcomes**	N = 4,655,183	N = 892,484	
Primary outcome	*No. of Events (%)*		
Overdose	318 (0.007)	123 (0.014)	2.22 (1.80 - 2.73)
Secondary outcomes			
Respiratory complications	12,674 (0.27)	2,503 (0.28)	1.18 (1.13 - 1.23)
Unspecified adverse effects of opioids	3,080 (0.07)	1,027 (0.12)	1.93 (1.80 - 2.07)
Composite outcome	14,787 (0.32)	3,187 (0.36)	1.28 (1.23 - 1.33)

CI denotes confidence interval. Composite outcome included opioid overdose, respiratory complications, or unspecified adverse effects of opioids.

© 2020 Bykov K et al. *JAMA Network Open.*

eTable 6. Primary and Sensitivity Analyses for Secondary Outcomes

Outcome /Analysis	Adjusted Hazard Ratios (95% CI)
**Respiratory complications**	
Primary analysis[a]	1.68 (1.59-1.78)
Intention-to-treat	1.56 (1.48-1.65)
No exposure carry-over[b]	1.63 (1.54-1.73)
Follow-up from postoperative day 1[c]	2.08 (1.92-2.25)
**Unspecified adverse effects of opioids**	
Primary analysis[a]	1.77 (1.61-1.93)
Intention-to-treat	1.62 (1.48-1.76)
No exposure carry-over[b]	1.74 (1.58-1.92)
Follow-up from postoperative day 1[c]	2.36 (2.09-2.67)
**Composite outcome**	
Primary analysis[a]	1.70 (1.62-1.79)
Intention-to-treat	1.57 (1.50-1.65)
No exposure carry-over[b]	1.66 (1.57-1.75)
Follow-up from postoperative day 1[c]	2.15 (2.00-2.30)

CI denotes confidence interval

[a] Follow-up started on the day of surgery, patients were considered exposed on the day of and through the day following a treatment charge and were censored on deviation from the initial treatment regimen.

[b] Patients were considered exposed only on the day of a treatment charge.

[c] Follow-up started on postoperative day 1. Patients with naloxone charge on the day of surgery were excluded; propensity scores were re-estimated and the population was re-weighted.

© 2020 Bykov K et al. *JAMA Network Open.*

eFigure 1. Propensity Score Distribution Before and After Trimming and Weighting

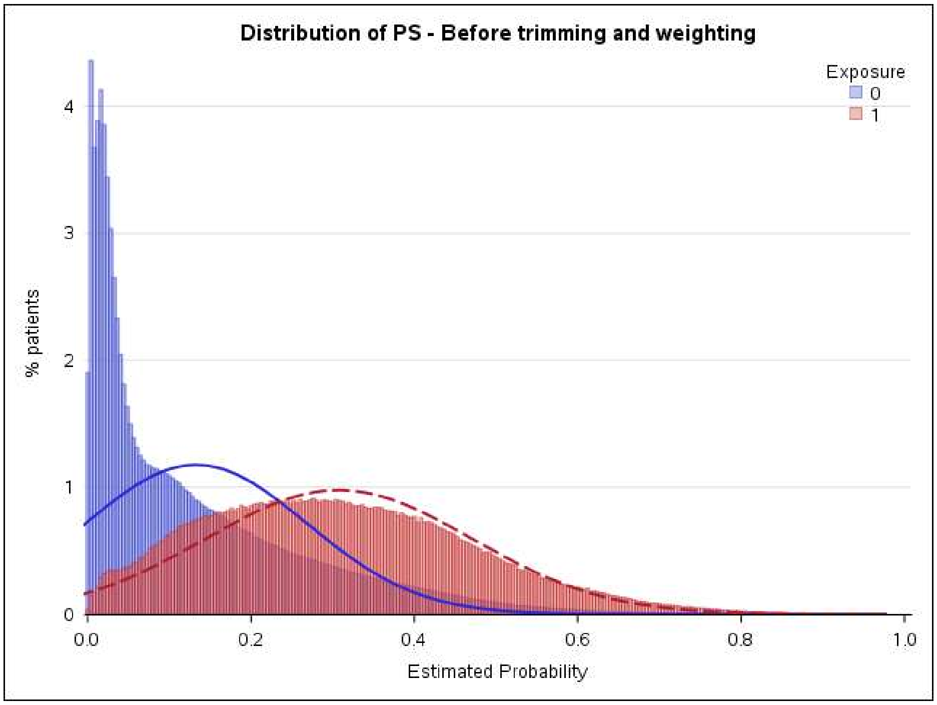

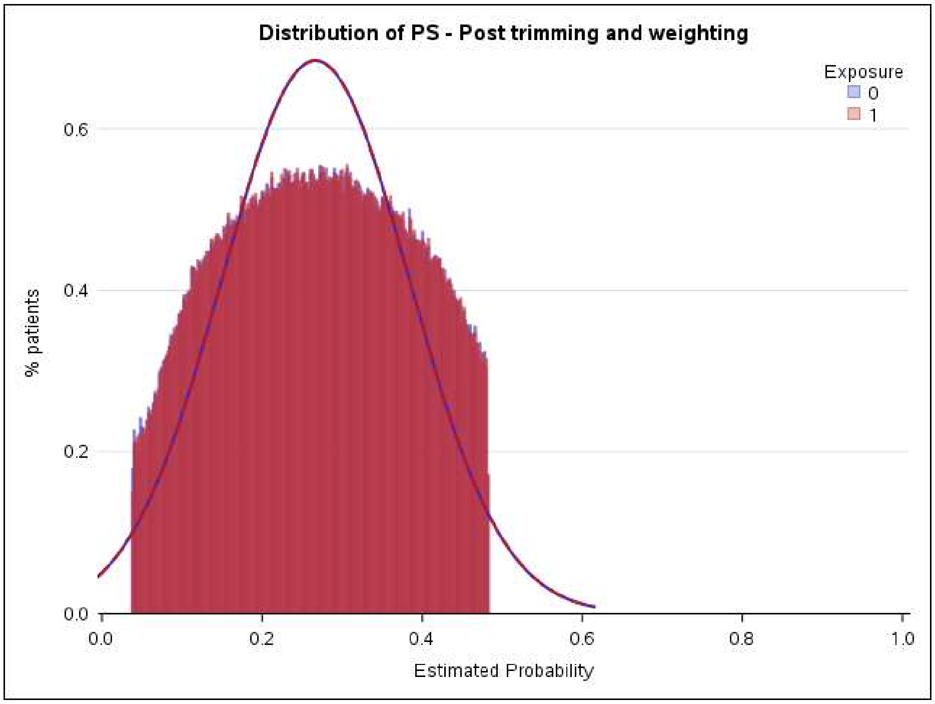

Exposure: 0 – opioids only; 1 – gabapentinoids + opioids; PS – propensity score

Number of patients trimmed with PS ≤ 2.5th percentile in the gabapentinoid-exposed: 1,536,324 opioids-only patients and 22,312 patients on gabapentinoids + opioids.

Number of patients trimmed with PS ≥ 97.5th percentile in the reference group (opioids only): 116,379 opioids-only patients and 132,789 patients on gabapentinoids + opioids.

© 2020 Bykov K et al. *JAMA Network Open.*

eFigure 2. Associations Between Exposure to Gabapentinoids and Opioids and the Outcome of Respiratory Complications Among Subgroups

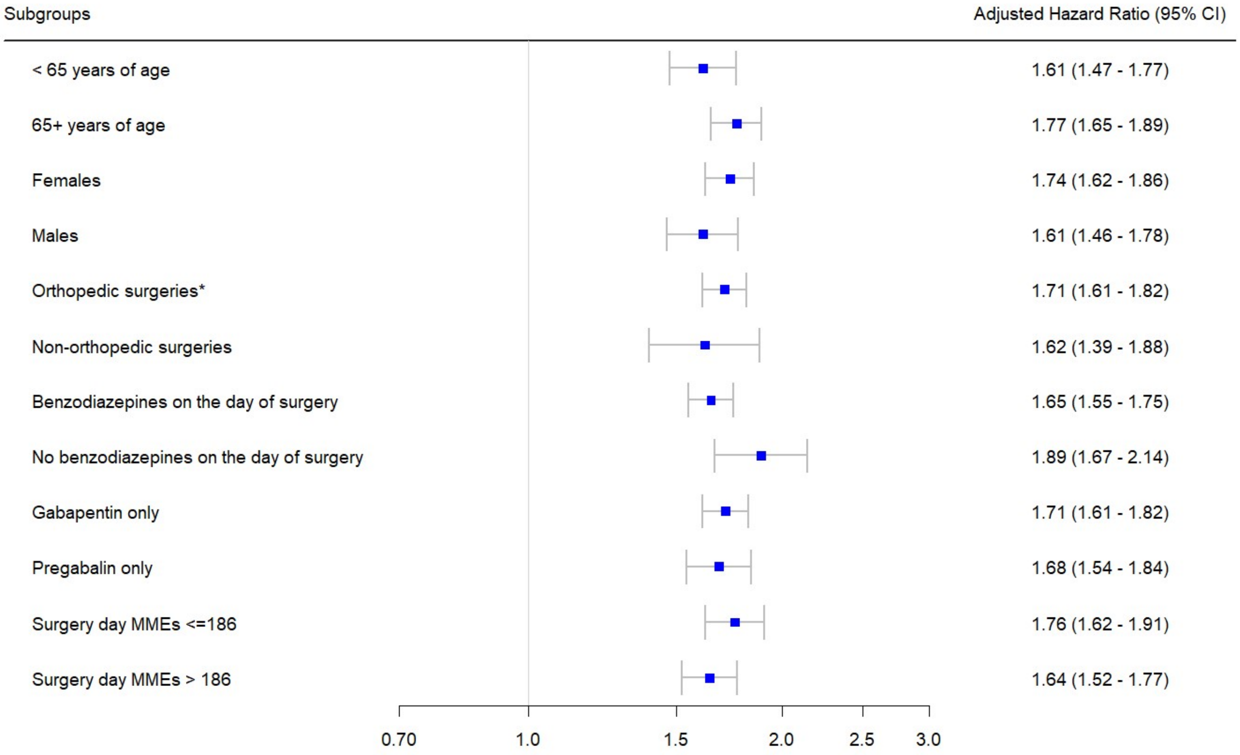

*orthopedic surgeries included hip arthroplasty, knee arthroplasty, laminectomy or spinal fusion, and a surgery for hip fracture or dislocation. MMEs denote morphine milligram equivalents.

© 2020 Bykov K et al. *JAMA Network Open.*

eFigure 3. Associations Between Exposure to Gabapentinoids and Opioids and the Outcome of Unspecified Adverse Effects of Opioids Among Subgroups

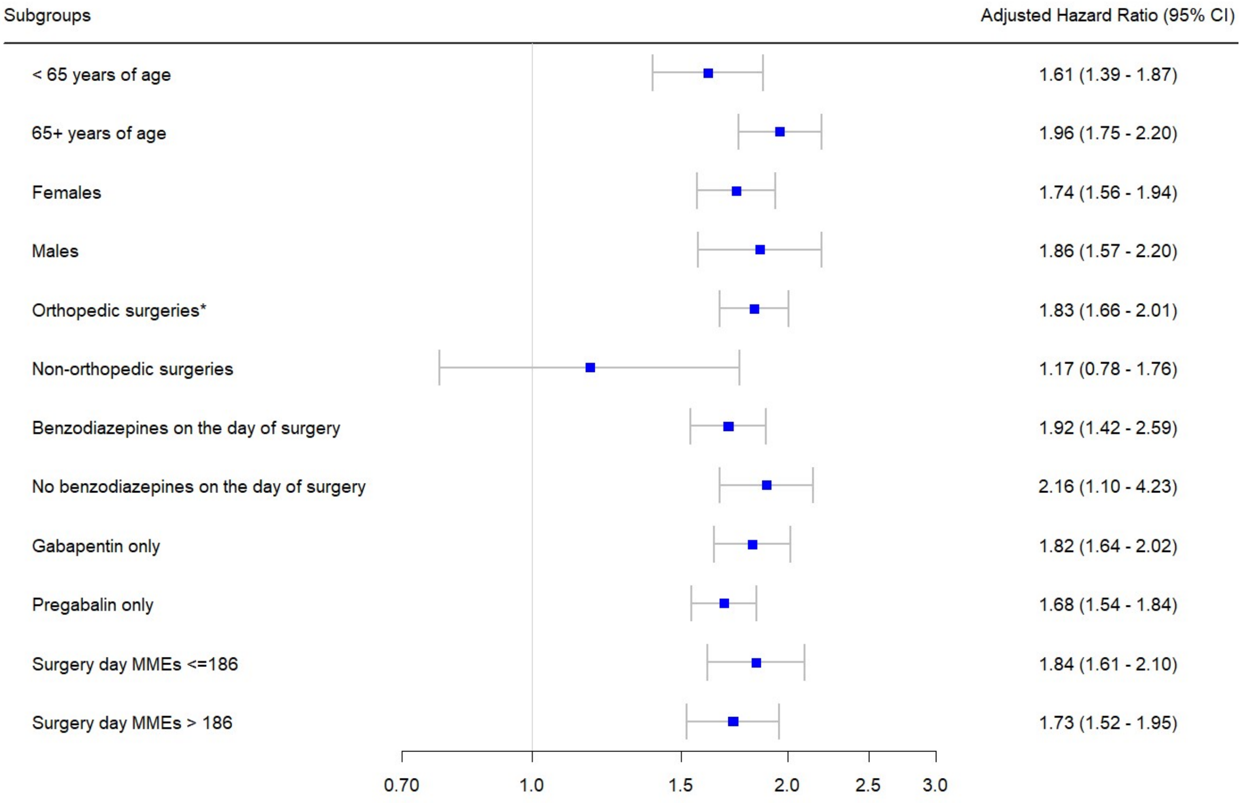

*orthopedic surgeries included hip arthroplasty, knee arthroplasty, laminectomy or spinal fusion, and a surgery for hip fracture or dislocation. MMEs denote morphine milligram equivalents.

© 2020 Bykov K et al. *JAMA Network Open.*

eFigure 4. Associations Between Exposure to Gabapentinoids and Opioids and the Composite Outcome of Overdose, Respiratory Complications, and Unspecified Adverse Effects of Opioids Among Subgroups

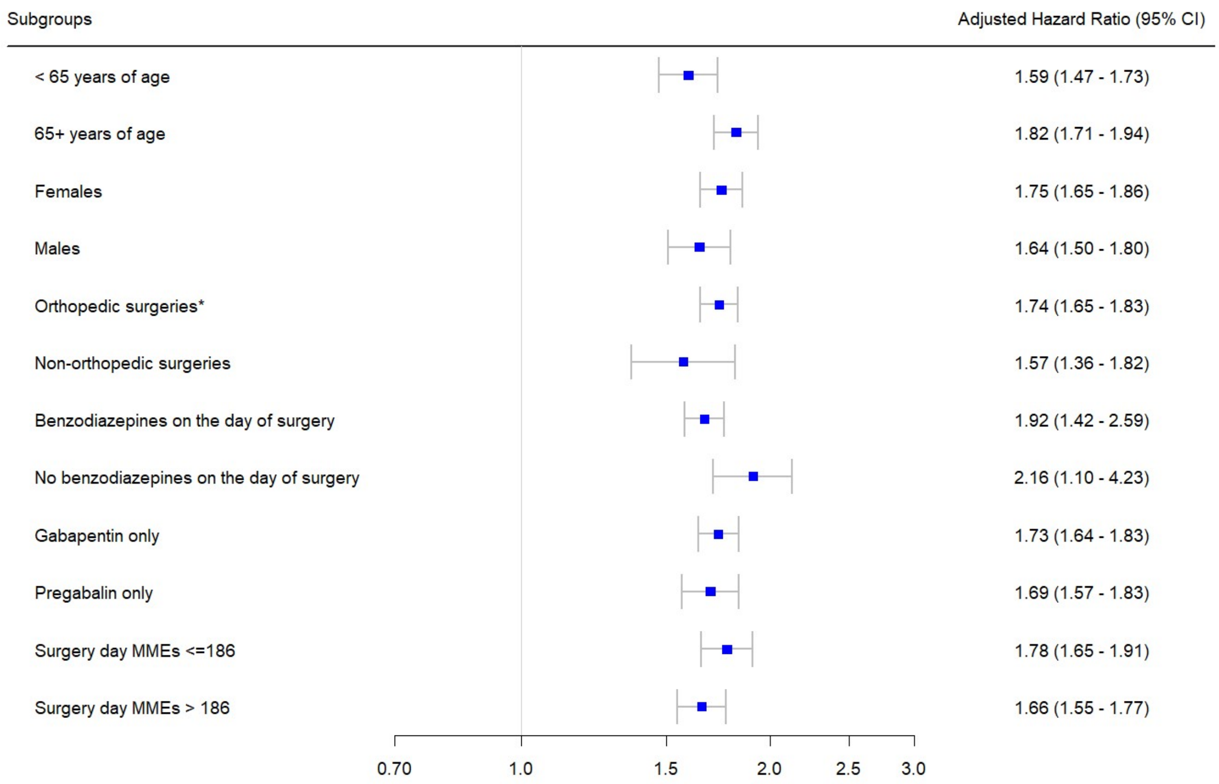

*orthopedic surgeries included hip arthroplasty, knee arthroplasty, laminectomy or spinal fusion, and a surgery for hip fracture or dislocation. MMEs denote morphine milligram equivalents.

© 2020 Bykov K et al. *JAMA Network Open.*
